# Supplementary material for: The NHE3 Inhibitor Tenapanor Prevents Intestinal Obstructions in CFTR-Deleted Mice
Source: Int J Mol Sci. 2022 Sep 1;23(17):9993. doi: 10.3390/ijms23179993 (PMC9456459; doi:10.3390/ijms23179993)
Supplement: Supplementary file 1 [file ijms-23-09993-s001.zip › ijms-1874758- supplementary.pdf]

Supplementary Materials

Table S1. List of Primers.

Gene	Forward	Reverse
Rps9	AAGCACATCGACTTCTCCC	ACAATCCTCCAGTTCAGCC
Ly6g	GAGAGGAAGTTTTATCTGTGCAGCC	TCAGGTGGGACCCCAATACA
Muc1	GCAGTCCTCAGTGGCACCTC	CACCGTGGGCTACTGGAGAG
Muc2	CCTGAAGACTGTCGTGCTGT	GGGTAGGGTCACCTCCATCT
Mcp1	CTGCTGTTCACAGTTGCCG	GCACAGACCTCTCTCTTGAGC
**Gene**	**Company / Catalog number**	
Tnfα	Quantitect # QT00104006	
Il1β	Quantitect # QT01048355	
Enacα	Quantitect # QT00198289	
Enacβ	Quantitect # QT00104335	
Enacγ	Quantitect # QT00126168	

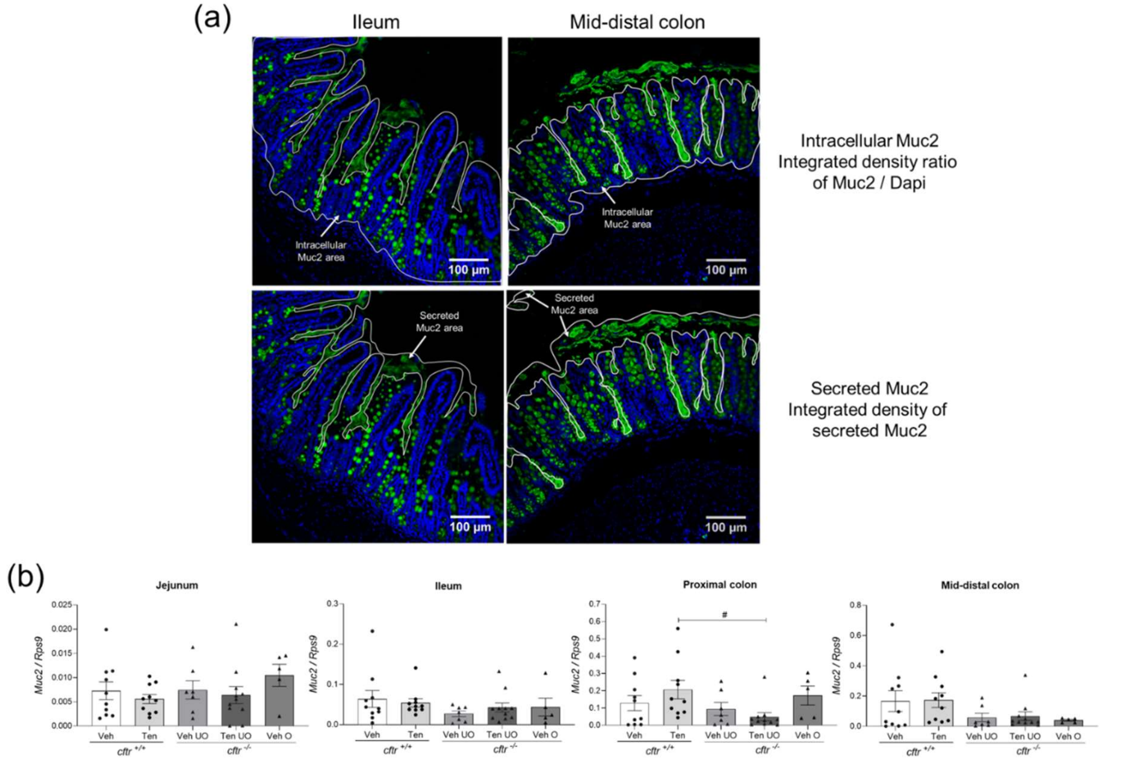

Figure S1. (a) Example of how the intra- and luminal mucus compartments were delineated and subsequently assessed. Both the intracellular, as well as the luminal secreted Muc2 associated immunofluorescent intensity [1] was measured and related to the Dapi associated immunofluorescence. (b) *Muc2* mRNA expression. Each dot/ triangle represents one mouse. Veh – vehicle, Ten – tenapanor, UO – unobstructed, O – obstructed. Data is represented as mean ± SEM.

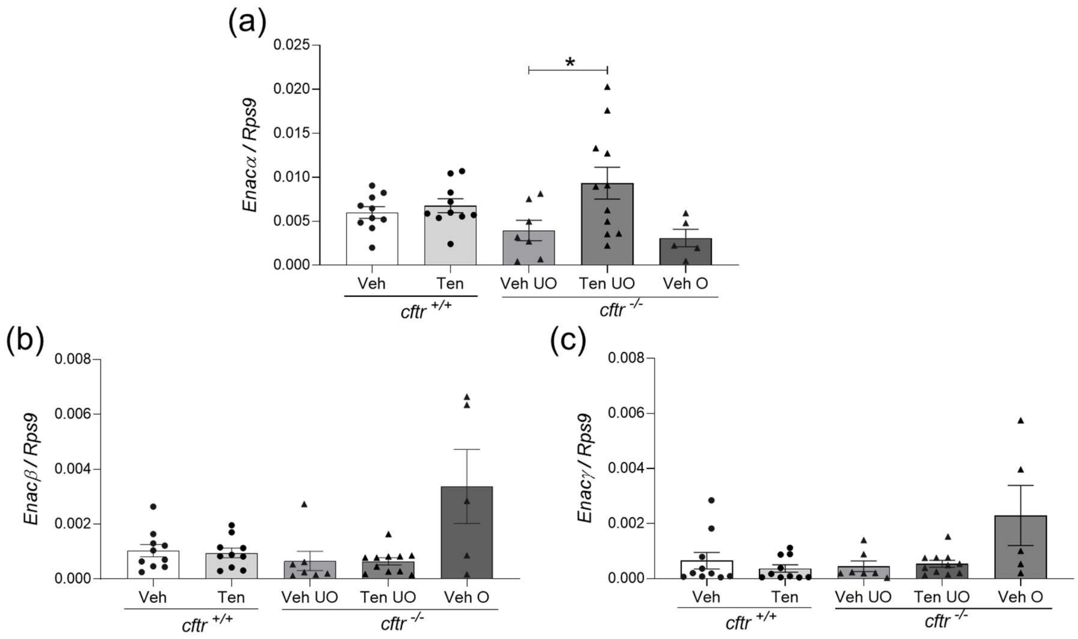

Figure S2. mRNA expression of (a) *Enacα*, (b) *Enacβ*, and (c) *Enacγ* in the mid-distal colon. Each dot / triangle represents one mouse. Veh – vehicle, Ten – tenapanor, UO – unobstructed, O – obstructed. Data is represented as mean ± SEM.

Reference

1. Honda, S.; Loher, P.; Morichika, K.; Shigematsu, M.; Kawamura, T.; Kirino, Y., et al. Increasing cell density globally enhances the biogenesis of Piwi-interacting RNAs in Bombyx mori germ cells. *Scientific Reports* **2017**, *7*, 4110.
